# Cross subkey side channel analysis based on small samples

**DOI:** 10.1038/s41598-022-10279-9

**Published:** 2022-04-15

**Authors:** Fanliang Hu, Huanyu Wang, Junnian Wang

**Affiliations:** 1 School of Physics and Electronic Science, Hunan University of Science and Technology, Xiangtan, 411201 China; 2 School of EECS, KTH Royal Institute of Technology, Stockholm, Sweden

**Keywords:** Computer science, Information technology

## Abstract

The majority of recently demonstrated Deep-Learning Side-Channel Analysis (DLSCA) use neural networks trained on a segment of traces containing operations only related to the target subkey. However, when the size of the training set is limited, as in this paper with only 5K power traces, the deep learning (DL) model cannot effectively learn the internal features of the data due to insufficient training data. In this paper, we propose a cross-subkey training approach that acts as a trace augmentation. We train deep-learning models not only on a segment of traces containing the SBox operation of the target subkey of AES-128 but also on segments for other 15 subkeys. Experimental results show that the accuracy of the subkey combination training model is $28.20\%$ higher than that of the individual subkey training model on traces captured in the microcontroller implementation of the STM32F3 with AES-128. And validation is performed on two additional publicly available datasets. At the same time, the number of traces that need to be captured when the model is trained is greatly reduced, demonstrating the effectiveness and practicality of the method.

## Introduction

Side-Channel Analysis (SCA) have become a realistic threat to implementations of cryptographic algorithms, such as Advanced Encryption Standard (AES)^1^. Even theoretically secure cryptography may be broken since the encryption has to run in hardware or software at some point to actually do things. There might be some unintentional physical leakage during the execution of a cryptographic algorithm, such as the power consumed^2,3^ by the victim device. By utilizing the unintentional physical leakage^4–6^, it is possible for SCA to bypass the theoretical strength of cryptographic algorithms and to recover the key. This is particularly threatening since once the secret key is leaked, the ciphertext can be decrypted and the signature can be forged.

Recently, DL^7^ techniques have made significant breakthroughs in image classification^8^ and natural language processing (NLP)^9^. Because DL models are good at finding relevance from within the data, more and more SCA researchers are applying DL techniques in SCA. Currently, there are many studies demonstrating that DLSCA is more effective than traditional SCA^10–12^. In^13^, Huanyu et al. evaluated the impact of SCA for the diversity of target chips. In^14^, Maikel et al. investigate the impact of changes in the loss function on SCA and introduce multiple loss functions to improve the efficiency of SCA. In^15^, Yoo-Seung et al. evaluated the black-box hardware AES engine using the DL models. The above researchers usually choose the tracking of target subkeys and operations associated with target subkeys as the subject of their experiments.

Most of these existing deep-learning based attacks use a divide-and-conquer strategy to recover a 128-bit secret key of AES-128, in which the 128-bit key *K* is divided into 8-bit parts $k_i \in \mathscr {K}=\{ 0,\,1,\, \ldots ,\,\,255\}$, called *subkeys*, for $i \in \left\{ {1,\;2,\; \ldots ,16} \right\}$. We use $\mathscr {K}$ to denote the set of all possible subkey candidates. Afterwards, each subkey $k_i$ is recovered independently by using the deep-learning models trained on traces only related to a specific subkey $k_i$.

However, deep learning models always have the problem of not being able to effectively learn features within the data when there are not enough traces to train. A common solution for this is data augmentation, which is to use modified version of existing data to expand the training set. In SCA, a trace segment leaked by an operation related to the $i_{th}$ subkey $k_i$ could be used as an augmenting trace for another subkey $k_j$, with the same operation and the same input. In some implementations of AES-128, instructions are computed sequentially and procedures are executed byte-by-byte. This means if two identical operations have the same input data, for example, two SBox substitutions in the first round of AES, the resulting power consumption or electromagnetic emission could be similar. Probably this is noticed before but the potential benefit of training models on traces for multiple subkeys has not been fully explored.

In this paper, we propose a cross-subkey training approach that uses multiple subkeys rather than a single subkey to build models with better fitting capacity. By adding a certain amount of traces which are related to the non-targets subkeys, the profiling data set can be considered as a data augmentation for the traces of the target subkey. Our current results show that (1) the number of traces included in the training sets is kept constant, and the effectiveness of cross-subkey training is verified by varying the proportion of target and non-target subkeys in the training sets; (2) adding traces of non-target subkeys to the target subkey training sets expand the training set and can effectively improve the efficiency of the side-channel analysis by twofold. The method is also validated on the home-made dataset AES_STM32 and the publicly available datasets AES_XMEGA, AES_GPU.

## Background

This section first reviews AES-128. Afterwards, we briefly introduces deep learning and how to apply deep learning to side-channel analysis. For a broader introduction for deep learning, see^7^. Finally, the three evaluation metrics used in this paper are presented.

### AES-128

AES^1^ is one of the most widely used symmetric cryptographic algorithm standardized by NIST in FIPS 197 and included in ISO/IEC 18033-3. AES-128 is a subset of AES which takes a 128-bit key *K* to encrypt a 128-bit block of plaintext *P*, and the output is a 128-bit block of ciphertext *C*. AES-128 contains 10 encryption rounds in total and except the last round, each round repeats 4 steps sequentially: *SubBytes*, *ShiftRows*, *MixColumns* and *AddRoundKey*. The final round does not contain *MixColumns*. In our experiment, the mode of operation is set to Electronic Codebook (ECB) mode, which first divides the message into blocks and each block is encrypted separately. The *SubBytes* procedure is a non-linear substitution which maps an 8-bit input to an 8-bit output by using the Substitution Box (SBox).

An attack point for side-channel analysis is a selected intermediate state which can be used to describe the power consumed by the victim device during the execution of AES. The selection of attack point is affected by known input data (e.g. plaintext, ciphertext) and physical measurements (e.g. power consumption, EM emissions, timing). Two common points of attack are the first round of SBox output and the last round of SBox input of the AES algorithm. An appropriate attack point will lead to a more efficient attack.

### Deep-learning side-channel attack

Deep learning is a subset of machine learning^16^ that uses deep neural networks to learn from experience and understand the input data in terms of a hierarchy of concepts. Since deep-learning techniques are good at extracting features in raw data^7,17,18^, deep-learning based SCA become several orders of magnitude more effective than the traditional cryptanalysis. A typical deep-learning side-channel attack can be divided into two stages.

At the profiling stage, the attacker aims to use the deep-learning model to learn a leakage profile by using a large set of power traces $\mathscr {T} = \{ {{\text {T}}_1}{\text {,}}{{\text {T}}_2},\ldots ,{{\text {T}}_m}\}$ captured from the profiling device, where *m* is the number of traces in the training set. Each trace $T_i$ is labeled by the data processed at the attack point $l({\text {T}}_i)\in L$, where $L = \{ 0,\,1,\, \ldots ,\,\,255\}$, which can be used to derive the subkey by using some known input (e.g. the plaintext, ciphertext). The process of building a neural network can be viewed as a mapping $\mathscr {N}: \mathbb {R}^m \rightarrow \mathbb {I}^{\left| L \right| }$ and the output is a *score* vector $S=\mathscr {N}({\text {T}})\in \mathbb {I}^{\left| L \right| }$. The element $s_j$ with value *j* in *S* represents the probability that $l({\text {T}})=j$.

At the attack stage, the attacker uses the trained deep-learning model to classify traces captured from the victim device and obtain the score vector. The attacker can find the $i_{th}$ subkey ${k_i}=j$ which has the largest probability in *S*. We use ${k_i^*}$ to denote the real subkey. Once ${k_i}= {k_i^*}\;$, the subkey is recovered successfully. To quantify the classification error of the neural network, we use the cross-entropy^16^ as the loss function and the optimizer is set to RMSprop (Root Mean Square prop).

1 $$\begin{aligned} {k_i} = \mathop {\arg \max }\limits _{0 \le j \le 255} \;{{\tilde{s}}_j}. \end{aligned}$$

### Evaluation metrics

#### Accuracy

Model accuracy is defined as the probability of a model achieving correct classification results on a testing set. As one of the most commonly used model evaluation metrics in machine learning, model is used to characterise a model’s ability to classify data. An increase in model accuracy accuracy indicates that the backpropagation algorithm’s optimization of the weights and bias parameters gradually converges to the correct values, and the model gradually converges to the optimal model. The loss of a model characterises the degree of deviation between a model’s predicted and actual values. The smaller the loss, the closer the model’s prediction is to the actual value. The loss function used in this experiment is the *Categorical Crossentropy*. The formula for the accuracy of the model is:

2 $$\begin{aligned} acc(X_{attack})=\frac{|\{x_{i}\in X_{attack}\}\tilde{k}|}{X_{attack}}. \end{aligned}$$

where $X_{attack}$ denotes the test dataset, $x_{i}$ denotes the *i*th power trace in that dataset, $\tilde{k}$ denotes the calculation result, and $x_{i}\in X_{attack}$ is the set when the guess keys are all equal to the correct key. The model’s accuracy is the ratio of the number of power traces when the guessed key is equal to the correct key to the number of power traces in all the testing sets.

#### PGE and key rank

However, when traces are noisy^19^, it might be difficult for the model to predict the key with a single traces. In that case, partial guessing entropy (PGE) becomes a more suitable evaluation criterion. PGE indicates the mean rank of the real subkey sorted by the predicted probabilities of all possible subkeys. During the attack stage, we use the trained model to classify traces from the testing set and obtain the probabilities of different keys for each trace. For trace $x_{i} \in X_{attack}$, the obtained probability matrix is denoted as $P_{i}=[p_{i,1}, p_{i,2}, \ldots , p_{i,255}]$, where $p_{i,j}$ in $P_{i}$ is the predicted probability of *k*=*j* for trace $x_{i}$. Where $P_{i}$ is the correct Key Rank, which is usually used as an evaluation criterion for datasets with better signal-to-noise ratios, as the number of traces used to recover the correct key for datasets with higher signal-to-noise ratios is usually in the single digits, and using the Key Rank provides a more intuitive evaluation of the results. The lower the number of traces in the Key Rank, the better the model.

Afterwards, we apply an element-wise multiplication for all $P_{i}$ to obtain a cumulative probability:

3 $$\begin{aligned} \mathbf{P} =\prod _{i=1}^{m}P_{i}=[\mathbf{P} _{0},\mathbf{P} _{1},\ldots ,\mathbf{P} _{255}] \end{aligned}$$

where *m* is the number of traces we used for classification. Then, PGE can be represented as the averaged rank of real key $k^{*}$ sorted by $\mathbf{P}$.

## Cross-subkey attack

**Figure 1:**
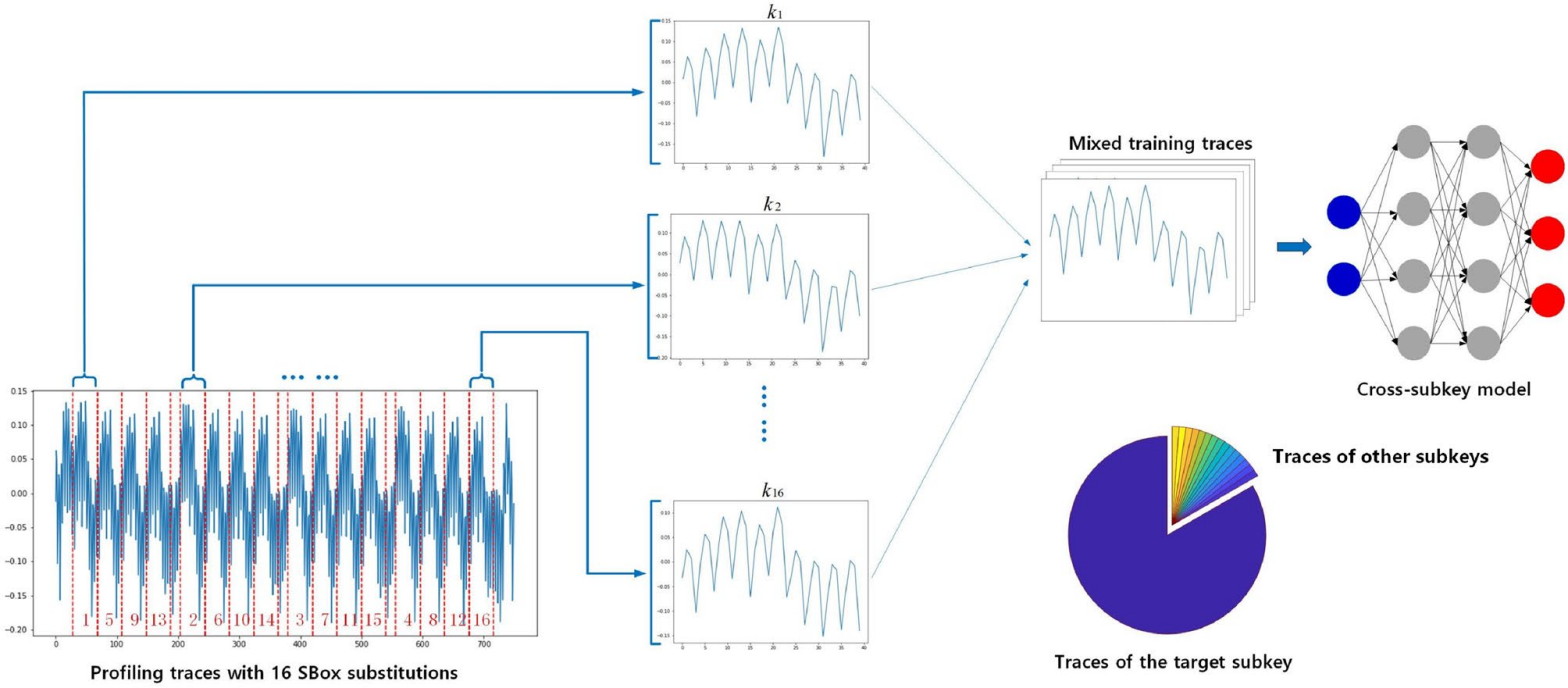
An overview of how the cross-subkey model is trained on the mixed profiling set.

Figure 1 shows an overview of how the cross-subkey model is trained, where the collaborative use of different subkeys provides more feature information to the model, providing a better fit to the target subkey.

### Composition of power traces

Power based side-channel analysis utilize the fact that the power consumed during the execution of the encryption process by the victim device might be different according to the different input data and different operations. Therefore, the most interesting parts of a power consumption trace can be defined as a data-dependent component $\mathscr {P}_{data}$ and an operation-dependent component $\mathscr {P}_{op}$. Besides, using the same device to repeat the same operation with the same input data will also consume different amount of power for every repetition because of the electronic noise component $\mathscr {P}_{noise}$. Meanwhile, the switching activities of the transistors which are independent from the input data can generate a constant amount of power consumption, which is called the constant component $\mathscr {P}_{const}$. Thus, each point of a power trace can be modeled as the sum of these components^3^.

4 $$\begin{aligned} \mathscr {P}_{total}=\mathscr {P}_{data}+\mathscr {P}_{op}+\mathscr {P}_{noise}+\mathscr {P}_{const} \end{aligned}$$

### Trace augmentation

Deep-learning techniques have performed remarkably well on many side-channel attack scenarios. However, deep learning models are inadequately trained to measure and always suffer from an inability to effectively learn features within the data. Unfortunately, many attackers may not have access to big profiling data, for instance, attackers may not have a full control to the profiling device and can only capture a limited amount of traces. One data-level solution to the problem of limited training data is data augmentation^20^, which aims to use the additional synthetically modified traces to act as a regularizer and helps enhance the fit when training models in the context of side-channel analysis.

**Figure 2:**
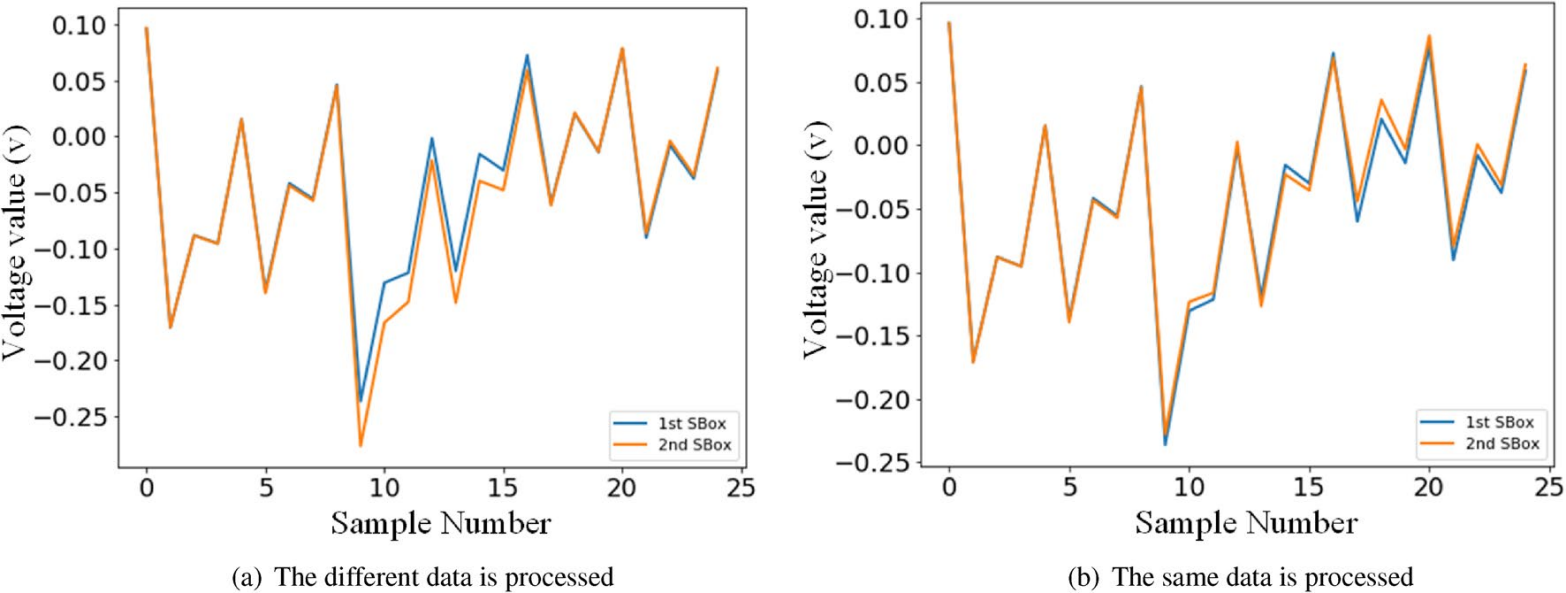
Power traces captured from an 8-bit microcontroller implementation of AES, which represent the first SBox and the second SBox operations in the first round. Traces look very similar if the same data is processed.

In software implementations of AES, leakage is time-dependent since instructions are carried out one by one^21^. This leads to a generally accepted approach for the attack to against software implementation of AES, which is to build a leakage profile between traces and the target subkey. Typically for the 8-bit microcontrollers and microprocessors, the encryption is implemented byte by byte. If the same data is processed by two SBox substitutions, power traces of these two operations could be similar since the the data-dependent components and operation-dependent components in formula 4 are the same. Figure 2 shows power traces captured from an 8-bit microcontroller implementation of AES, which represent the first SBox and the second SBox operations in the first round. One can see that power traces look very similar if the same data is processed by two SBox substitutions. So we could use a small amount of traces related to the non-target subkeys as a regularizer for the training set which contains traces only for the target subkey. It is a data augmentation for a specific subkey to build the model with a better fitting capacity.

### Cross-subkey model training

As shown in Fig. 1, a trace which contains 16 SBox computations of the first round is first divided into 16 sub-traces. The $i_{th}$ sub-trace is labeled by $l_i$ which represents the output of the $i_{th}$ SBox procedure, with $p_i$ denotes the $i_{th}$ byte of the plaintext.

5 $$\begin{aligned} l_i = SBox(p_i \oplus k_i) \end{aligned}$$

At the profiling stage, traces are divided into 16 sub-traces by analyzing the Point of Interest (POI), and each sub-trace is labeled by the corresponding SBox output. Generally, to recover the $i_{th}$ subkey, attackers train deep-learning models on sub-traces which are labeled by the $i_{th}$ SBox output. In the cross-subkey training, we go to one step further by adding a small amount sub-traces which represent the other 15 SBox operations into the training set.

We divided the experiment into two parts (notice: the number of training sets in this paper is 5*K*):

- **Verifying the validity of cross-subkey training** (total training set 5*K* constant). We define the proportion of subtraces of the target subkey to the total training set as $x \in [ 1, 16 ]$. Thus the proportion of other subkeys in the training set is $16 - x$. The other 15 subkeys are average distributed in the training set.
- **Applying cross-subkey training** (total training set is increased by 5*K* at a time). We use all the power traces of the target subkey (5*K* in this paper) for training, and add an equal number of power traces (5*K*) to the training set at a time as the number of target power traces, which are provided by the other 15 subkeys. The training set is thus $5K \times y (y \in [ 1, 16 ])$, where $5K \times (y-1)$ is equally distributed in the training set by the other 15 subkeys.

## Experimental results

In this section, we first introduce the DL model structure. Afterwards, the training setup is presented. Finally we show the experimental results of the cross-subkey side channel analysis method on three datasets. We use the $\rho$-test as a leak detection method^22^ to find the point of interest (POI) for each subkey. The power consumption model used in this paper is the identity model^13^.

### Model structure

**Figure 3:**
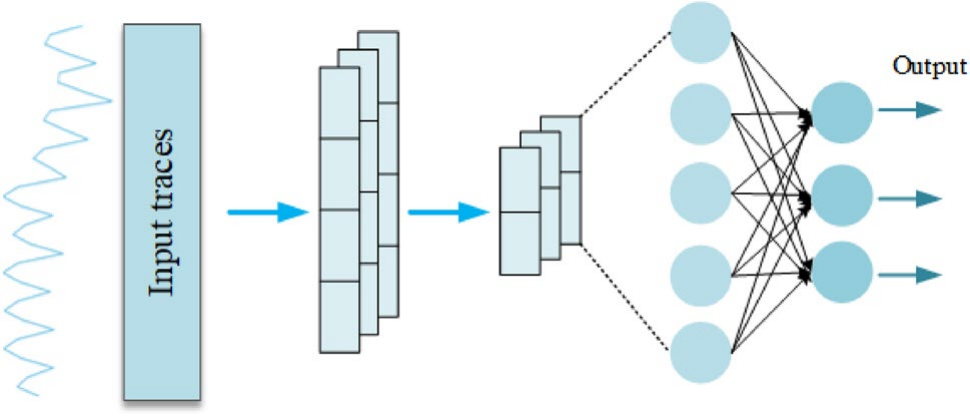
DL model structure.

The structure of the network model used in this work is shown by Fig. 3. After passing through the Input Layer, the traces are connected to a Convolutional Layer with a step size of 5 and a neuron count of 16. After passing through an Average Pooling Layer with a pooling step of 3, there are expanded by Flatten and then connected to two Dense Layers with 256 neurons. The last of these Dense Layers is activated by Softmax and is used to generate 256 output predictions. The Activation Functions of the other layers in the network model are all with Rectified Linear Unit (Relu).

### Training setup

We divide the experiment into two parts the first part in order to demonstrate that the inclusion of sub-traces of non-target subkeys positively influences the training of the model, and the second part for the application of the cross-subkey approach to the experiment.

#### Part I

We know that data augmentation increases the amount of training data by adding minor alterations to the existing training traces. However, too many alterations in the training set may confuse the neural network. So to find the optimal amount of augmenting traces in the training set becomes a realistic problem. Thus, for each database, we build 16 different training sets, which contains different amount of augmenting traces to train 16 deep-learning models. Figure 1 shows an example of how these training sets are built. We call these training sets from $set_1$ to $set_{16}$. Suppose the database contains *x* traces for training and we divide each trace to 16 segments as shown in Fig. 1, which are related to 16 subkeys separately. So the total number of trace segments should be $16 \times x$. To train the model for the target subkey, the training set is composed of *x* target subkey segments and *y* other-subkey segments. Segments of 15 non-target subkeys are equally distributed in all training sets. From $set_1$ to $set_{16}$, the ratio of the target-subkey segments to all segments is defined by $\frac{x}{x+y} \in \{ \frac{1}{{16}},\frac{2}{{16}},\ldots \frac{{16}}{{16}}\}$, in which $set_{16}$ denotes the set without trace augmentation. The corresponding trained models are denoted by $M_{1}, M_{2}, \ldots , M_{16}$.

#### Part II

In image classification, data enhancement methods are often used such as cropping, rotating, flipping, deflating and shifting^23^. These methods are essentially a series of changes to the original data in order to expand the number of training sets on which the models are trained. In the Part I, we do not change the number of training sets on which the models are trained. The main work in this part is to use all the traces of the target subkey and expand the training set with other subtraces of non-target subkeys for the purpose of data augmentation. Assuming that the database contains x training traces, similar to the work in Part I, we will also train 16 models. The training sets of 16 models are denoted by $\tilde{s}et_{1}$ to $\tilde{s}et_{16}$. The amount of data in $\tilde{s}et_{y} (y \in [ 1, 16 ])$ is $x \times y$, where $(16 - y) \times x$ is equally distributed by the sub-traces of non-target subkeys. $\tilde{s}et_{1}$ denotes all traces of the target subkey *x* (no sub-traces of other subkeys), and $\tilde{s}et_{16}$ denotes all traces of all subkeys $16 \times x$. The corresponding trained models are denoted by $\tilde{M}_{1}, \tilde{M}_{2}, \ldots , \tilde{M}_{16}$.

### Results on software AES-128 implementation on STM32F3 (AES_STM32)

The first dataset is captured by a ChipWhisperer-Lite^24^ device at a sampling frequency of 40MHz. The experimental target cryptographic board is the CW308T-STM32F3, and the target cryptographic chip is the Arm Cortex M4, which runs the cryptographic algorithm TinyAES-128. The encryption mode of operation is the Electric Code Book (ECB) mode.

**Figure 4:**
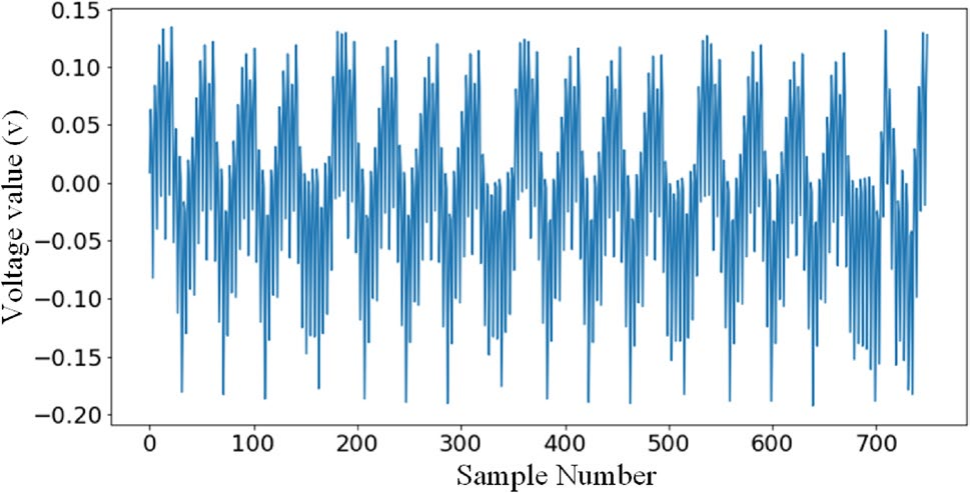
Waveform of the first round traces of the STM32F3 implementation of TintAES-128 (AES_STM32).

For the first round of the AES algorithm 11*K* power traces are captured as the data used for the experiments. Of these, 6*K* uses random plaintexts and random keys, 5*K* is used as the training set and 1*K* is used as the validation set. The remaining 5*K* are used as the testing set for the experiments using fixed-key random plaintexts. Each power trace has 750 sampling points and contains all *SubBytes* from the first round. This is shown by Fig. 4. We call this homemade dataset AES_STM32 in brief.

**Figure 5:**
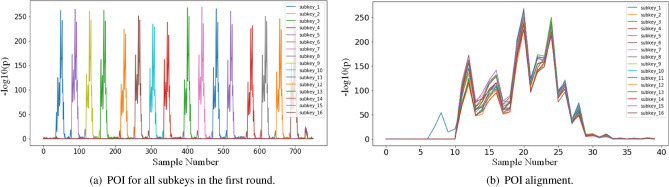
The result of all subkey POI and the result of POI alignment (AES_STM32).

#### Results

*Experiment I* We first used the $\rho$-test to detect the POI of each subkey, as shown in Fig. 5a. The POI of each subkey in this dataset corresponds to 40 sampling points on the trace. Specifically, the trace segment for the first SBox operation is $\mathrm{{[28: 68]}}$ (The $1_{st}$ subkey as the target subkey). Figure 5b shows how we allowed to synchronise segments for different bytes of the subkeys. In this experiment we generated 16 training sets, called $set_1$, $set_2$, ..., $set_{16}$, based on the training method in Part I. Each training set contains 5*K* traces, with 1*K* of data for the target subkey as the validation set, which will be saved during model training when the model is at its highest accuracy in the validation set. The testing set is the one containing 5*K* traces of the target subkey, and we also tested the other subkeys, which also contained 5*K* traces of the corresponding subkeys. Afterwards, model $M_1$, $M_2$, ..., $M_{16}$ is trained on the corresponding training set respectively. The training batch_size are set to 256 and the maximum number of epochs is 500 and the learning rate is 0.0005. Since the optimiser RMSprop is random in updating parameters, we have trained each model 10 times and taken the mean value as the experimental result. Table.1 shows the accuracies of the 16 models on the full testing set of subkey. where $M_{i} (i \in [1, 16])$ denote the models and $S_{i} (i \in [1, 16])$ denote the testing set of different subkeys, e.g. the first column in the first row shows the accuracy of $M_{1}$ on the testing set of the first subkey (accuracy figures are in percentages, with the % omitted at the end).

We found that model $M_{15}$ had the highest accuracy on the testing set of the first subkey. Because the training set of model $M_{16}$ is the full trace of the first subkey, a model trained by means of cross-subkey will be $6.52\%$ more accurate than a model trained traditionally on a one-to-one approach. Next, we show the results of the trace number increase in the training set.

**Table 1:** Classification accuracies of 16 DL models on a testing sets of 16 subkeys (No change in the number of traces in the training set).

	$M_{1}$	$M_{2}$	$M_{3}$	$M_{4}$	$M_{5}$	$M_{6}$	$M_{7}$	$M_{8}$	$M_{9}$	$M_{10}$	$M_{11}$	$M_{12}$	$M_{13}$	$M_{14}$	$M_{15}$	$M_{16}$
$S_{1}$	5.86	8.74	9.56	11.01	11.78	12.77	11.96	13.91	13.83	14.00	14.13	14.69	16.70	18.46	21.27	14.75
$S_{2}$	5.07	5.16	5.14	4.73	4.09	5.69	4.56	3.52	3.12	3.47	3.14	2.77	2.70	2.47	2.00	0.34
$S_{3}$	6.31	6.55	6.53	6.40	6.12	6.48	4.30	5.07	5.45	4.17	4.37	4.49	4.22	3.02	2.33	0.36
$S_{4}$	4.87	5.81	6.09	5.72	5.18	5.19	4.42	4.19	4.21	4.34	2.94	3.90	3.00	2.22	2.11	0.36
$S_{5}$	7.62	8.15	8.46	8.77	8.17	8.41	8.43	6.38	7.02	6.44	6.20	5.35	4.16	3.53	4.10	1.95
$S_{6}$	5.93	7.28	6.76	6.82	6.09	6.61	6.68	5.41	6.11	5.49	5.07	4.63	4.29	3.37	2.80	1.05
$S_{7}$	7.99	8.92	7.43	8.44	7.90	8.38	7.67	6.41	6.23	6.09	5.66	5.50	5.40	3.61	3.93	0.72
$S_{8}$	6.22	7.40	6.31	7.15	7.00	7.16	6.86	6.24	6.38	5.16	4.49	5.14	4.24	2.62	2.90	0.88
$S_{9}$	5.22	6.72	6.13	6.60	6.51	5.85	7.37	5.97	6.20	5.99	7.03	5.80	6.95	7.34	5.78	1.63
$S_{10}$	4.08	4.27	4.66	3.88	3.92	3.89	4.38	3.93	3.84	3.43	3.95	3.12	3.76	3.19	1.82	0.51
$S_{11}$	4.18	5.42	6.52	4.78	4.67	4.91	4.77	4.44	4.27	3.30	3.96	4.07	4.55	3.88	3.04	0.77
$S_{12}$	3.79	4.93	5.30	4.96	4.09	4.11	5.15	4.68	4.25	3.74	3.88	3.34	3.74	3.82	3.21	0.84
$S_{13}$	7.12	7.73	6.85	7.09	7.09	7.05	7.58	6.55	6.37	5.66	5.16	4.71	5.08	4.37	3.60	0.71
$S_{14}$	5.11	6.01	5.30	5.43	5.89	5.19	5.91	5.36	4.71	3.87	3.52	3.76	2.81	2.53	1.59	0.35
$S_{15}$	5.77	6.91	6.52	6.53	6.16	6.67	6.16	5.79	4.91	4.46	4.01	4.70	3.35	3.55	2.58	0.50
$S_{16}$	5.38	5.83	5.84	6.05	5.78	5.43	6.46	6.49	5.24	4.71	3.65	4.12	2.96	2.21	2.19	0.76

*Experiment II *Again in this subsection the first subkey is used as the target subkey. In contrast to *Experiment I* the number of training sets for each model is increasing when training the cross-subkey model, with the training set being increased by 5*K* traces at a time, and these 5*K* traces being equally distributed among the sub-traces of the other non-target subkeys. Where the training set for $\tilde{M}_{1}$ is all the traces of the first subkey and the training set for $\tilde{M}_{16}$ is all the traces of all subkeys. The models $\tilde{M}_{i} (i \in [1, 16])$ is then trained on the corresponding dataset. The other hyperparameters are the same as *Experiment I* . Finally each model is trained 10 times and the results on the testing sets of different subkeys are taken as the mean value for the experimental results. Table.2 shows the accuracy of the 16 models on the testing set of all subkeys, where $\tilde{M}_{i}$ denotes the models and $S_{i} (i \in [1, 16])$ denotes the testing set of different subkeys (accuracy figures are in percentages, with the % omitted at the end).

We found that model $\tilde{M}_{10}$ had the highest accuracy on the testing set of the first subkey. It is $28.20\%$ more accurate than the traditional one-to-one trained model $\tilde{M}_{1}$ on the testing set of the first subkey.

**Table 2:** Classification accuracies of 16 DL models on a testing sets of 16 subkeys (Increasing number of traces in the training sets).

	$\tilde{M}_{1}$	$\tilde{M}_{2}$	$\tilde{M}_{3}$	$\tilde{M}_{4}$	$\tilde{M}_{5}$	$\tilde{M}_{6}$	$\tilde{M}_{7}$	$\tilde{M}_{8}$	$\tilde{M}_{9}$	$\tilde{M}_{10}$	$\tilde{M}_{11}$	$\tilde{M}_{12}$	$\tilde{M}_{13}$	$\tilde{M}_{14}$	$\tilde{M}_{15}$	$\tilde{M}_{16}$
$S_{1}$	14.75	21.56	27.16	28.79	31.52	31.47	34.78	32.55	38.53	42.95	37.37	37.39	37.63	36.78	39.08	39.85
$S_{2}$	0.35	4.76	9.09	11.87	14.53	15.94	18.26	18.81	19.12	21.81	23.29	23.54	24.80	25.40	27.66	25.93
$S_{3}$	0.36	8.51	12.49	14.15	14.90	15.53	19.07	18.97	20.01	23.08	23.42	23.92	25.67	26.14	28.50	26.81
$S_{4}$	0.36	9.05	12.79	13.21	17.42	16.69	19.76	18.97	21.78	23.88	26.54	23.98	26.13	27.75	28.63	23.72
$S_{5}$	1.94	11.87	14.30	17.13	18.29	20.73	25.59	23.23	27.39	30.20	27.02	32.56	30.54	31.55	33.88	35.64
$S_{6}$	1.01	9.29	12.59	11.50	14.14	16.27	19.16	19.22	19.98	19.70	23.54	23.85	25.02	28.15	25.87	29.85
$S_{7}$	0.72	9.73	13.04	12.75	13.22	13.74	16.97	15.79	18.17	18.47	22.88	23.15	22.60	24.07	23.09	26.41
$S_{8}$	0.88	9.99	11.78	12.76	12.62	14.01	15.95	16.12	15.96	17.21	20.17	21.25	19.54	22.54	20.39	23.23
$S_{9}$	1.63	7.82	11.05	10.15	14.07	14.26	17.01	17.29	21.22	21.43	21.84	23.19	25.18	24.42	27.91	31.51
$S_{10}$	0.51	5.90	8.01	8.57	11.38	9.62	13.19	11.88	13.20	15.25	14.42	13.48	15.96	14.69	18.18	19.11
$S_{11}$	0.75	6.73	8.59	9.87	13.02	12.30	15.92	14.51	17.44	17.78	17.38	17.77	20.81	16.94	22.61	22.76
$S_{12}$	0.84	6.68	8.76	10.96	14.54	14.39	18.81	17.48	20.81	21.16	20.77	22.13	24.22	21.71	27.06	26.44
$S_{13}$	0.71	8.06	13.25	17.11	20.66	20.61	27.53	25.78	29.17	31.85	31.66	33.89	33.30	33.62	34.25	37.10
$S_{14}$	0.28	7.22	11.56	14.28	16.60	16.24	21.85	19.31	21.68	23.34	24.86	23.41	26.35	25.83	26.51	28.52
$S_{15}$	0.50	7.38	12.51	13.42	15.02	15.23	20.98	19.04	21.68	22.94	23.98	24.71	26.01	24.45	26.94	28.14
$S_{16}$	0.76	8.47	12.28	14.98	16.28	18.00	21.66	21.69	24.06	25.03	25.49	28.08	28.01	27.31	28.60	28.07

This is because classification accuracy partially reflects the effectiveness of the models on SCA. Next, we evaluated ${M}_{i}$ and $\tilde{M}_{i}$ on the testing set $S_{1}$ using Key Rank and PGE. The results are shown in Table.3. Because of the higher AES_STM32 signal-to-noise ratio, the higher classification accuracy of the DL models on the testing set $S_{1}$ and the lower number of traces needed to recover the correct key, for this dataset mainly Key Rank < 5 is used for the experimental comparison (the larger the number of traces with Key Rank < 5, the more efficient the DL models are at SCA). The final prediction result of ${M}_{15}$ on the testing set has 650 more traces than the prediction result of ${M}_{16}$ on the testing set Key Rank, and the prediction result of $\tilde{M}_{10}$ on the testing set $S_{1}$ has 2064 more traces than the prediction result of $\tilde{M}_{1}$ on the testing set $S_{1}$ Key Rank (where ${M}_{16}$ and $\tilde{M}_{1}$ are trained with the same training set).

**Table 3:** Results of Key Rank < 5 and PGE for $M_i$ and $\tilde{M}_{i}$ on testing set $S_{1}$ (AES_STM32).

DL model	$M_{1}$	$M_{2}$	$M_{3}$	$M_{4}$	$M_{5}$	$M_{6}$	$M_{7}$	$M_{8}$	$M_{9}$	$M_{10}$	$M_{11}$	$M_{12}$	$M_{13}$	$M_{14}$	$M_{15}$	$M_{16}$
Key Rank	843	1237	1327	1579	1615	1691	1697	1911	1886	1931	1887	1891	2179	2341	2746	2096
PGE	17	13	10	10	9	9	8	8	8	8	6	7	5	4	2	4

DL model	$\tilde{M}_{1}$	$\tilde{M}_{2}$	$\tilde{M}_{3}$	$\tilde{M}_{4}$	$\tilde{M}_{5}$	$\tilde{M}_{6}$	$\tilde{M}_{7}$	$\tilde{M}_{8}$	$\tilde{M}_{9}$	$\tilde{M}_{10}$	$\tilde{M}_{11}$	$\tilde{M}_{12}$	$\tilde{M}_{13}$	$\tilde{M}_{14}$	$\tilde{M}_{15}$	$\tilde{M}_{16}$
Key Rank	2096	2685	3113	3249	3383	3355	3523	3369	3727	4160	3741	3752	3689	3661	3775	3836
PGE	4	3	2	2	2	2	2	2	2	1	2	2	2	3	2	2

Next, we validate the method on two well-known publicly available datasets.

### Results on software AES-128 implementation on ATXMEGA128D4 (AES_XMEGA)

**Figure 6:**
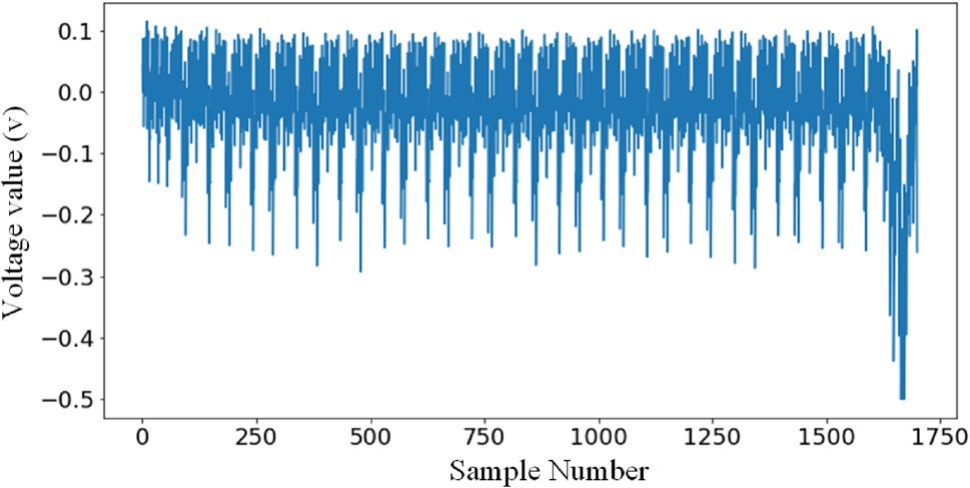
Waveform of the first round traces of the ATXMEGA128D4 implementation of TintAES-128 (AES_XMEGA).

The second dataset is captured using an 8-bit ATMEL microcontroller, the ATXMEGA128D4, and all the power traces generated during the encryption process are extracted using chipwhisperer to form this paper’s dataset, with the encryption mode being TinyAES-128’s electrical codebook (ECB) mode. The training, validation and testing sets of this dataset are set up in the same way as the first dataset. We call this dataset AES$\_$XMEGA in brief. Each power trace has 1700 sampling points and contains all *SubBytes* from the first round. This is shown by Fig. 6. Specific information on this dataset can be found in Literature^13^.

#### Results

**Figure 7:**
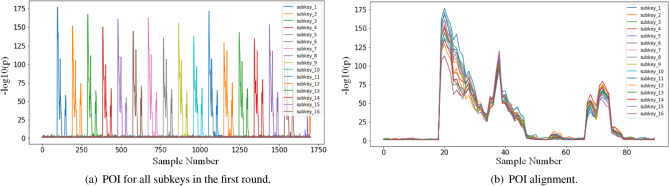
The result of all subkey POI and the result of POI alignment (AES_XMEGA).

The $\rho$-test is first used to locate the POI of the 16 subkeys on the traces, as shown in Fig. 7a. Each subkey leakage interval contains 90 sample points. Our target subkey is the first byte of the SBox output, which corresponds to a leakage interval of [858: 948]. Figure 7b shows how we allowed to synchronise segments for different bytes of the subkeys. The other experimental configurations are identical to the first dataset. During the training of the DL models, we used the RMSprop optimizer with a learning rate of 0.001. The mini-batch size is 256 and the maximum iterative epoch is 500. Next, the DL models are trained on a training sets that don’t change the number of traces contained in the training sets, which is denoted $M_{i}$. Finally, the DL models are trained on an increasing number of traces contained in the training sets, which is denoted $\tilde{M}_{i}$.

Table 4 shows the classification accuracies (accuracy figures are in percentages, with the % omitted at the end), Key Rank and PEG of the DL models trained with a constant number of traces in the training sets ($M_i$) and the DL models trained with a training sets with an increasing number of traces in the training sets ($\tilde{M}_{i}$) on the testing set $S_{1}$ for the first subkey. Since the target subkey is the first subkey, we only show the classification accuracies, Key Rank < 5 and PGE of the DL models on the testing set $S_{1}$ for the first subkey, and the training process for the other subkeys is the same as for the target subkey.

**Table 4:** Classification accuracy, Key Rank < 5 and PGE of $M_i$ and $\tilde{M}_{i}$ on the testing set $S_{1}$ (AES_XMEGA).

DL model	$M_{1}$	$M_{2}$	$M_{3}$	$M_{4}$	$M_{5}$	$M_{6}$	$M_{7}$	$M_{8}$	$M_{9}$	$M_{10}$	$M_{11}$	$M_{12}$	$M_{13}$	$M_{14}$	$M_{15}$	$M_{16}$
Accuracy	28.31	31.65	36.78	40.20	43.07	45.39	47.09	42.49	48.55	51.23	50.41	47.31	49.64	54.34	50.72	47.86
Key Rank	2749	3067	3458	3641	3811	3887	3953	3777	3974	4142	4058	3934	4058	4311	4064	3980
PGE	6	5	4	4	4	4	3	3	3	2	2	2	3	1	1	2

DL model	$\tilde{M}_{1}$	$\tilde{M}_{2}$	$\tilde{M}_{3}$	$\tilde{M}_{4}$	$\tilde{M}_{5}$	$\tilde{M}_{6}$	$\tilde{M}_{7}$	$\tilde{M}_{8}$	$\tilde{M}_{9}$	$\tilde{M}_{10}$	$\tilde{M}_{11}$	$\tilde{M}_{12}$	$\tilde{M}_{13}$	$\tilde{M}_{14}$	$\tilde{M}_{15}$	$\tilde{M}_{16}$
Accuracy	47.86	62.07	77.32	83.89	86.94	86.97	91.20	89.84	89.85	91.86	91.46	94.01	92.00	92.60	92.97	90.47
Key Rank	3980	4434	4775	4873	4939	4935	4970	4948	4960	4974	4978	4996	4976	4987	4973	4951
PGE	2	1	1	1	1	1	1	1	1	1	1	1	1	1	1	1

The results show that when the size of the training sets don’t change, $M_{14}$ trained with cross-subkey have a 6.48% higher classification accuracy than $M_{16}$ trained with the original training set, the number of Key Rank increases by 331 and the number of PGE decreases by 1. When the size of the training sets are increasing, $\tilde{M}_{12}$ trained with cross-subkey have 46.15% higher classification accuracy than $\tilde{M}_{1}$ trained with the original training set, the number of Key Ranks increased by 1016 and the number of PGE decreased by 1 (where ${M}_{16}$ and $\tilde{M}_{1}$ are trained with the same training set).

### Results on AES-128 Parallel implementation on GPU (AES_GPU)

**Figure 8:**
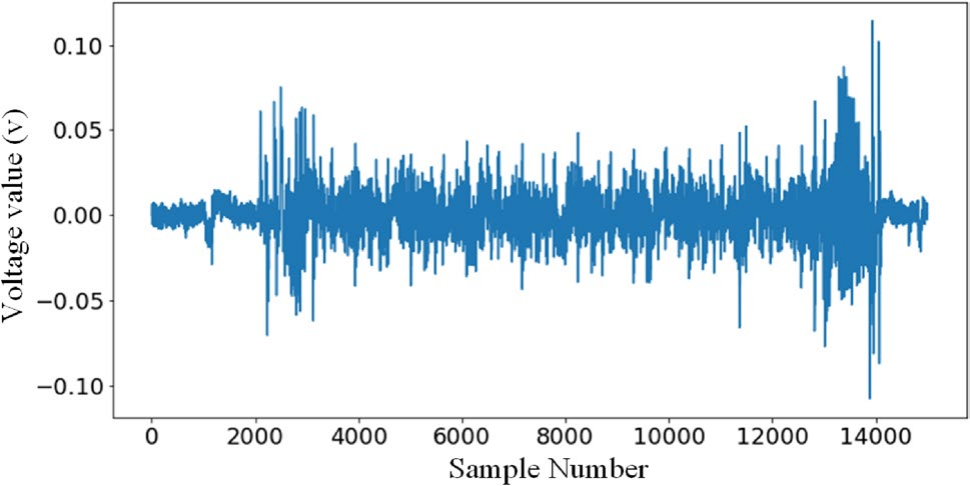
Waveform of the last round traces of the NVIDIA GeForce GT620 graphics card implementation of TintAES-128 (AES_GPU).

The third dataset is an NVIDIA GeForce GT620 graphics card (GPU) connected to the host with a PCIe bus. The AES parallel implementation (32 threads in a warp) and trace acquisition details are stated in^25^. The dataset has 34, 511 traces for analysis and 5, 000 traces for attacks. Because the paper uses a small sample dataset, we set the training sets, validation set and testing set for this dataset to be the same as the first dataset. We call this dataset AES$\_$GPU in brief. Each power trace contains 15, 001 sampling points as shown in Fig. 8.

#### Results

**Figure 9:**
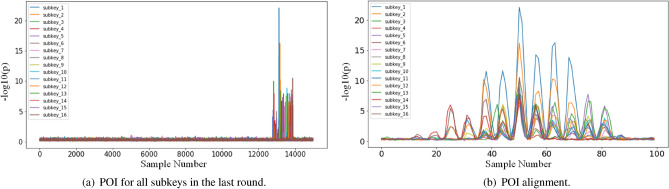
The result of all subkey POI and the result of POI alignment (AES_GPU).

The $\rho$-test is first used to locate the POI of the 16 subkeys on the traces, as shown in Fig. 9a. Each subkey leakage interval contains 100 sample points. Our target subkey is the $16_{th}$ byte of the SBox output, which corresponds to a leakage interval of [13081: 13181]. Figure 9b shows how we allowed to synchronise segments for different bytes of the subkeys. The other experimental configurations are identical to the first dataset. During the training of the DL models, we used the RMSprop optimizer with a learning rate of 0.0001. The mini-batch size is 256 and the maximum iterative epoch is 500. Next, the DL models are trained on a training sets that don’t change the number of traces contained in the training sets, which is denoted $M_{i}$. Finally, the DL models are trained on an increasing number of traces contained in the training sets, which is denoted $\tilde{M}_{i}$.

Table 5 shows the classification accuracies (accuracy figures are in percentages, with the % omitted at the end), Key Rank < 5 and PGE (cannot recover the correct key replace with “-”) of the DL models trained with a constant number of traces in the training sets ($M_i$) and the DL models trained with a training sets with an increasing number of traces in the training sets ($\tilde{M}_{i}$) on the testing set $S_{16}$ for the $16_{th}$ subkey. Since the target subkey is the $16_{th}$ subkey, we only show the classification accuracies of the DL models on the testing set $S_{16}$ for the $16_{th}$ subkey, and the training process for the other subkeys is the same as for the target subkey.

**Table 5:** Classification accuracy, Key Rank < 5 and PGE of $M_i$ and $\tilde{M}_{i}$ on the testing set $S_{16}$ (AES_GPU).

DL model	$M_{1}$	$M_{2}$	$M_{3}$	$M_{4}$	$M_{5}$	$M_{6}$	$M_{7}$	$M_{8}$	$M_{9}$	$M_{10}$	$M_{11}$	$M_{12}$	$M_{13}$	$M_{14}$	$M_{15}$	$M_{16}$
Accuracy	0.49	0.43	0.60	0.64	0.82	0.88	0.81	0.79	1.04	1.12	1.07	0.99	0.86	1.10	1.47	1.19
Key Rank	93	89	97	105	145	127	138	156	181	201	170	196	180	167	354	210
PGE	–	–	3211	3012	2321	2411	2891	3366	1942	1465	1652	1765	2545	1607	671	865

DL model	$\tilde{M}_{1}$	$\tilde{M}_{2}$	$\tilde{M}_{3}$	$\tilde{M}_{4}$	$\tilde{M}_{5}$	$\tilde{M}_{6}$	$\tilde{M}_{7}$	$\tilde{M}_{8}$	$\tilde{M}_{9}$	$\tilde{M}_{10}$	$\tilde{M}_{11}$	$\tilde{M}_{12}$	$\tilde{M}_{13}$	$\tilde{M}_{14}$	$\tilde{M}_{15}$	$\tilde{M}_{16}$
Accuracy	1.19	1.05	0.90	1.29	1.18	1.16	1.20	1.18	1.18	1.18	1.25	1.54	1.81	1.52	1.37	1.14
Key Rank	210	182	185	224	223	191	208	197	186	224	198	383	409	312	205	167
PGE	865	1047	1901	1424	1365	1497	1302	1170	865	852	799	560	331	597	745	962

The results show that when the size of the training sets don’t change, $M_{15}$ trained with cross-subkey have a 0.28% higher classification accuracy than $M_{16}$ trained with the original training set, the number of Key Rank increases by 144 and the number of PGE decreases by 194. When the size of the training sets are increasing, $\tilde{M}_{13}$ trained with cross-subkey have 0.67% higher classification accuracy than $\tilde{M}_{1}$ trained with the original training set, the number of Key Ranks increased by 199 and the number of PGE decreased by 534 (where ${M}_{16}$ and $\tilde{M}_{1}$ are trained with the same training set).

### Discussion

We set up two sets of experiments to validate on the homebrew dataset AES_STM32 and the public datasets AES_XMEGA, AES_GPU respectively. In Experiment I, the number of traces in the training set used when each model is trained is constant, and what is changed is the proportion of subtraces of the target subkey and subtraces of the non-target subkey in the training set. Because the model structure and hyperparameters are identical for the 16 models, only one independent variable, the training set, is used during the experiments. The experimental results show that by varying the proportion of target and non-target subkeys in the training sets (i.e. training the DL models using cross-subkey) when the size of the training set does not change, the final experimental results are improved in all three datasets. Because of the random nature of the iterative process of the parameters during the training of the neural network, we have repeated the training 10 times for each model and took the average accuracy, Key Rank and PGE of each DL model on the testing set with different subkeys as the experimental results.

Experiment I is designed to validate the effectiveness of the cross-subkey training model. Model $\tilde{M}_{1}$ is trained using the full trace of the target subkeys. Model $\tilde{M}_{i} (i \in [2, 16])$ is trained using a training set that is expanded with sub-traces of non-target subkeys. In AES_STM32, $\tilde{M}_{10}$ improved classification accuracy by 28.20% over $\tilde{M}_{1}$ on the testing set $S_{1}$, with an increase of 2064 traces for Key Rank < 5 and a decrease of 3 traces for PGE. In AES_XMEGA, $\tilde{M}_{12}$ improved classification accuracy over $\tilde{M}_{1}$ on the testing set $S_{1}$ by 46.15%, the number of Key Rank < 5 traces increased by 1016, and the number of PGE traces decreased by 1. In AES_GPU, $\tilde{M}_{13}$ improved classification accuracy over $\tilde{M}_{1}$ on the testing set $S_{16}$ by 0.67%, the number of Key Rank < 5 traces increased by 199, and the number of PGE traces decreased by 534. The results of Experiment II showed that by using the non-target subkeys traces to expand the training set obtained twofold better results than the model trained with the target subkeys.

Finally, when training the model, if a trace of a non-target subkey is added to the training set, the model is equally effective on the testing set of non-target subkeys. This result suggests that the traditional approach of one model recovering one subkey can be replaced by one model recovering all subkeys.

## Conclusion

In this paper, we propose a cross-subkey deep-learning side-channel analysis, which utilizes the additional synthetically modified power traces as a data augmentation to build models with a better fitting capability. Our results show that the accuracy, Key Rank and PGE of the models on the testing set can be improved by adding traces of other subkeys to the training set of the target subkeys when the traces of the capture are limited. This paper validates the effectiveness of the cross-subkey training models on the homebrew dataset AES_STM32 and the publicly available datasets AES_XMEGA, AES_GPU, but there are still many open rows for the links between different subkeys. As mentioned in the previous sections, there are many possible directions of research regarding the connections between different subkeys, which will ultimately bring more cohesion to the field and more confidence in the results obtained.
